# Research article Individual innovation from the perspective of nursing students: A qualitative study

**DOI:** 10.1186/s12912-023-01311-y

**Published:** 2023-05-15

**Authors:** Naval Heydari, Mahnaz Rakhshan, Camellia Torabizadeh, Ghasem Salimi

**Affiliations:** 1 Student Research Committee, Department of Nursing, School of Nursing and Midwifery, Shiraz University of Medical Sciences, Shiraz, Iran; 2 Community Based Psychiatric Care Research Center, Department of Nursing, School of Nursing and Midwifery, Shiraz University of Medical Sciences, Shiraz, Iran; 3 Department of Educational Administration and Planning, Faculty of Education and Psychology, Shiraz University, Shiraz, Iran

**Keywords:** Innovation, Innovativeness, Students, Nursing

## Abstract

**Background:**

One of the necessities of today’s world that prepares nursing students for their future professional roles is the concept of individual innovation. However, there is no clear definition of individual innovation in nursing. This study was designed and implemented with the aim of investigating the concept of individual innovation from the perspective of nursing students using qualitative content analysis.

**Methods:**

This qualitative study was conducted from September 2020 to May 2021 on 11 nursing students of one of the nursing schools in southern Iran. The participants were selected by purposive sampling method. Data were collected through semi-structured individual interviews. Data analysis was done using conventional content analysis and MAXQDA 2018.

**Results:**

Following data analysis, 662 initial codes were extracted forming 9 categories and three main themes. The themes included personal and professional dynamism, professional inventiveness, and the integration of innovation drivers.

**Conclusions:**

The concept of individual innovation in the nursing student included personal and professional dynamics and professional inventiveness. Individual innovation occurred through combination of innovation drivers. Managers and policy makers of nursing education can use the results of this to get acquainted with this concept and develop policies and guidelines to develop nursing students’ individual innovation. Through getting familiar with the concept of individual innovation, nursing students can try to flourish this characteristic in themselves.

**Supplementary Information:**

The online version contains supplementary material available at 10.1186/s12912-023-01311-y.

## Background

One of the necessities of today’s changing world is innovation [1]. Individual innovation refers to the ability to take risks in the face of newness, adapt and adopt, apply and tolerate, and experience new things [2]. Individual innovation means the desire to search and find new approaches to solve a problem using new and already existing resources [3]. Cerinšek and Dolinšek (2009) define individual innovation as “A person’s willingness to act and react in an innovative way to deal with events, problems or tasks that require innovative thinking and reactions and can occur in a specific context” [4].

In the recent decades, health care system reform and the clinical professional development have been proposed based on innovative roles for health care providers, of whom nurses have the most important roles [1]. The designation of 2009 as the Year of Innovation by the International Council of Nurses (ICN) aimed at increasing international competitiveness and developing the intellectual horizon of scientific institutions [5]. This designation highlights the importance of innovation in the field of nursing worldwide. On the other hand, some scientists have emphasized the transfer of creativity and innovation to nursing practice [6]. In this regard, Rogers has used innovation to describe the services provided by nurses in the future. She acknowledges that by understanding the components of innovation, it is possible to know how nursing can remain innovative and survive as a suitable and desirable profession [7]

The presence of innovative individuals in nursing profession improve patients’ access to care and promotes health [8]. For example, new smartphone apps were designed by innovators to guide healthcare staff, improve patient’s access to care, help people to monitor their health changes, and provide support to chronic patients [9]. Challenges of emerging technologies, cost adjustment, and quality of care require deep attention to the nature of individual innovation and what it is. In addition, it is important to know how innovative people think and see problems and how we can teach, strengthen, or suppress innovation [8]

A study was conducted by Kaya et al., (2015) in Turkey aiming at analyzing the concept of innovation in nursing. They stated that innovation was an important concept in nursing literature but with various definitions, indicating the lack of clarity on the definition of this concept in nursing. They concluded that the concept of innovation needed to be clarified to use a common terminology [10]. In a review study, Melnyk et al., (2009) in the United States also found that lack of familiarity with innovation was one of the major barriers to innovation in nursing schools [11]. Kamplyis et al., (2011) have also considered innovation as an essential part of education [12]. In nursing education, the use of innovation is emphasized to cultivate competent nurses and develop nursing knowledge and skills [13]. In order to benefit from innovation, nurses they should learn this concept from the beginning [10, 14]. Fostering individual innovation prepares students for their future professional role [15]. Based on a study in Turkey, through increasing the level of students’ individual innovation, their understanding of individual care also enhances [14]. Nursing students need to be innovative given that they are novice and inexperienced, they should deal with new and diverse situations in the clinical environment, and they are expected to identify their educational needs and respond to the care needs of patients [16, 17]. They should be able to implement innovation while caring for the patients and apply solutions that tailor to the needs and problems of each patient [14]. They are also expected to have an innovative way of thinking so that they can play their innovative role effectively [17]. Therefore, individual innovation seems an essential attribute for nursing students [2]. In Taiwan, creativity and innovation are considered as one of the main competencies of nursing students [18]. But in Iran, individual innovation is not considered as part of the process of formal education and professional socialization of nursing students [19]. Meanwhile, for several years, the need to modify the curriculum has become a common and important topic in the literature of nursing and health sciences in the world [20]. Despite the importance of fostering individual innovation in nursing students, limited studies have been conducted in this field merely describing the status of innovation and creativity in nursing students’, and few ones explored nursing students’ views toward innovation in nursing education [2, 6, 14]. Most of the conducted studies indicate a low level of individual innovation in nursing students and emphasize on paying attention to this issue [2, 14, 21]. Therefore, discovering the concept of individual innovation from the perspective of nursing students seems necessary.

Individual innovation is a concept that can also be influenced by contextual factors, such as culture, beliefs, and values governing society [22]. A qualitative study in Iran showed that innovation was sensitive to local and society conditions in entrepreneurship context [23]. Although there are descriptive studies investigated individual innovation in Iran, these studies were conducted on populations other than nurses, such as employees of gas corporation [24], school managers [25], graduate students in the School of Education, Psychology, Management, and Accounting of Allameh Tabatabai University [26]. The study on the employees of the gas corporation indicated the impact of interaction, leadership, communication, knowledge, integration, organizational support and motivation on individual innovation [24]. Study on school managers has shown that there is no relationship between thinking styles and individual innovation [25]. Study on the students of Allameh Tabatabai University has indicated a significant relationship between knowledge sharing and individual innovation [26]. To the best of our knowledge, no study has been conducted to describe and analyze the concept of individual innovation in nursing students in Iran. Marcati et al., (2008) distinguish between “general innovation”, the degree of openness to novelty, and “specific innovation”, which underlies the adoption of innovation in a specific domain [27], such as nursing. Innovation in nursing is used to generate information, protect people’s health, prevent diseases, and provide personalized care [10]. Therefore, the descriptions that have been proposed in general and globally for individual innovation may not be appropriate to the cultural and social context of the Iranian nursing students’ community, so it is necessary to analyze this concept in the context of Iranian culture and society.

To identify, describe, or discover a concept and its components, it is recommended to use a qualitative approach, because many ambiguities and possible related dimensions are spontaneously clarified through this approach, leading to the development and application of the concept in a particular field. Therefore, to understand the nature of nursing students’ individual innovation, it is necessary to use a qualitative approach considering that qualitative studies give the individuals the opportunity to express their views, values, and beliefs [28]. Therefore, this study was conducted with the aim of analyzing the concept of individual innovation from the perspective of nursing students using a qualitative approach.

## Methods

### Study design and participants

In this qualitative study, 11 nursing students of Shiraz University of Medical Sciences participated. This study lasted from September 2020 to May 2021. The participants were selected by purposive sampling method with maximum variation in terms of age, sex, marital status, and semester and degree, and among those who had experience or knowledge about the subject under study [28]. Inclusion criteria were as follows:

- Willingness to participate in the study.
- Having Bachelor of Science, Master of Science, or PhD in nursing.
- Scoring above 68 on Hurt et al. individual innovativeness scale (1977) [29]. to ensure that participants are innovative (because interviewees must have experience or knowledge of the subject under the study in qualitative interviews [28]). This scale is a one-dimensional scaling comprising of 20 items. It is scored based on a 5-point Likert scale. The scores range from 14 to 94. To calculate the total score, first the score of items 4, 6, 7, 10, 13, 15, 17, and 20 (step 1) and then the score of items 1, 2, 3, 5, 8, 9, 11, 12, 14, 16, 18, and 19 are added together (step 2). To obtain the total score, the following formula is used: Innovativeness score = 42 + total score for step 2 - total score for step 1. Individuals achieving a score above 68 are regarded as innovators. The construct validity of ISS is confirmed using Chi-square test. The reliability of ISS is 0.94 based on Nunnally’s technique (1967) [29, 30]. Before using ISS in the current study, the researchers translated the English version into Persian using the forward-backward translation method and validated the questionnaire in the community of Iranian nursing students. The qualitative face validity of the questionnaire was evaluated by obtaining the opinions of 15 nursing PhD students. The quantitative face validity of ISS was confirmed after calculating the impact score, which was above 1.5 for all items. In addition, 15 experts in nursing confirmed the qualitative content validity of ISS. Instrumentation and quantitative content validity of ISS was also confirmed by using the Lawshe’s method and calculating content validity ratio, which was between 0.6 and 1 for the items and 0.85 on average. The content validity index for the items was between 0.8 and 1 and was 0.91 on average. The construct validity was examined through exploratory factor analysis. Based on factor analysis and scree plot, three factors were extracted with eigenvalue > 1, which cumulatively explained 55.49% of the changes in the items. The reliability of the tool was also confirmed (Cronbach’s alpha coefficient = to 0.880). The stability of the ISS was assessed by calculating intraclass correlation coefficient (ICC) and confirmed considering a 95% confidence interval ranging from 0.894 to 0.976(ICC = 0.949).
- Having an experience of innovation and invention, being a member of the Talent Committee of the University, or being a member of Iran’s National Elites Foundation.

Exclusion criterion included the following:

- Withdrawing from the study during or after the interview.

### Data collection

After recruiting participants based on the inclusion criteria, the objectives of the study, interview method, and the time and place of the interview were explained to the participants. Then, informed consent was obtained from the participants. Data gathering was done through individual, in-depth, semi-structured interviews while ethical issues were considered. One of the classes of the School of Nursing and Midwifery, which was located in a quiet place, was considered for face-to-face interviews. However, due to the conditions caused by the COVID-19 pandemic and the absence of some students, a number of interviews were conducted via WhatsApp video call (5 participants) or telephone (3 participants due to the participant’s unwillingness to make video call). Three participants (4th semester female PhD student, 5th semester female undergraduate student and, 5th semester male PhD student) were interviewed twice due to the length of the interview and their request to continue the interview in the next session. Each interview session lasted between 25 and 55 min. Some of the questions raised included the following:

- What does individual innovation in nursing students mean in your view?
- What are the components of individual innovation in your view?
- What are your experiences of innovation as an innovator in nursing?

Interview improvement techniques, such as probing, giving examples, describing, using exploratory questions, summarizing the interviewee’s responses, active listening, and reflection of the participant’s speech, were used to improve the interview process [28]. Bracketing was also used and the interviewer tried not to interpret the interviewees’ responses. The interviews ended with summarizing the content, announcing the end or continuation of the interview in future meetings, and thanking the participant. Interviews were transcribed on the same day.

### Data analysis

Interviews and data analysis were performed simultaneously with a combination of manifest and latent content analysis and using conventional inductive content analysis approach. Similar to Graneheim and Lundman’s method, our analysis process was not linear and involved moving back and forth between the original text and related parts of the text. At first, the transcription was read word by word several times in order to get immersed and acquainted with the text. The analysis unit was then obtained by putting together the transcription obtained from the interview. Next, the text was divided into meaning units, summarized, and labeled by codes. Different codes were compared on the basis of similarities and differences and sorted into subcategories and categories. Categories were discussed and revised by the researchers, and reflection continued until agreement was reached on the final coding. Finally, the underlying meanings, which were the content of the categories, were formulated in the themes [31]. For data analysis, MAXQDA 2018 was used (distributed and developed by VERBI Software Company in Berlin, Germany).

After 14 semi-structured interviews with 11 students, no new themes were obtained and data saturation was confirmed by members of the research team.

### Rigor

To establish rigor of our qualitative study, 4 criteria proposed by Lincoln and Guba (1985) were considered, including credibility, dependability, confirmability, and transferability [32]. Previous experience of researchers in qualitative study, persistent and long-term engagement with nursing students, triangulation, member check (by 4 people including two PhD students and 2 undergraduate students), and review of the extracted codes and categories by experts added to the credibility of our study. On the other hand, the participants were selected accurately to provide a diversity of age and gender and based on Hurt and colleagues’ individual innovativeness scale. In addition, the research questions were varied to obtain a sufficient amount of information. In order to increase the dependability and confirmability of the study, an external observer (a female associate professor of nursing with a PhD in nursing education and 29 years of professional experience) accompanied the research team during the study examining the process of data collection and content analysis. In addition, the researchers described the study method in detail and provided the necessary information to her. Transferability was also ensured through a thorough description of the categories, characteristics of the participants, and methods of data collection and analysis.

## Results

The majority of the participants were female, single, and with the age range of 20 to 37 years old. The demographic characteristics of the 11 nursing students participated in the current study are shown in Table 1.

**Table 1:** Demographic characteristics of the participants and inclusion criteria

Participants	Gender	Age	Education level	Semester	Innovation score
1	Female	34	PhD	2	86
2	Female	34	PhD	4	70
3	Female	37	PhD	7	84
4	Female	21	Bachelor’s degree	5	69
5	Male	32	PhD	5	77
6	Male	22	Bachelor’s degree	5	77
7	Male	22	Bachelor’s degree	5	84
8	Female	20	Bachelor’s degree	6	86
9	Male	27	Master’s degree	4	94
10	Male	25	Master’s degree	5	72
11	Female	29	Master’s degree	1	81

Following data analysis, 662 initial codes were extracted forming 9 categories and three main themes. The themes included personal and professional dynamics, professional inventiveness, and the integration of innovation drivers (Table 2).

**Table 2:** Themes and categories resulting from data analysis

Themes	Categories	Examples of interview questions
Personal and professional dynamism	Dynamics Existing from self-made fence Structural and process change	What does individual innovation in nursing mean in your view? What are the components of individual innovation in nursing students in your view? Can you explain the concept of dynamism a little more?
Professional inventiveness	Intellectual inventions in nursing Practical inventions in nursing	What is the meaning of individual innovation in nursing students? What is your definition of individual innovation in nursing students? What are the dimensions of individual innovation in nursing students?
Integration of innovation drivers	Mental, cognitive, and psychological capabilities Internal stimuli External stimuli Support networks	What factors affect the individual innovation in a nursing student? What are the characteristics of an innovative nursing student? Can you explain the factors facilitating individual innovation in nursing students? Can you explain the barriers to individual innovation in nursing students? Can you explain about your experience of an innovative activity? In your experience, what factors are helpful, or what factors can facilitate or hinder innovation in nursing students? What are the solutions to promote individual innovation in nursing students?

### Personal and professional dynamism

From the participants’ point of view, personal and professional dynamism was one of the important components of innovation in a nursing student. The categories were dynamics, existing from self-made fence and structural and process change.

#### Dynamics

Many participants regarded dynamics, vitality, and movement as essential to innovation. The dynamics category itself included subcategories of individual dynamics and professional dynamics. The participants believed that innovators were people with dynamic personalities. They stated that the nursing system and organizations as well as clinical and educational organizations must be dynamic and lively in order to bring up an innovative student.

*I think an innovative person has a dynamic personality …… If people involved in education, from lecturers to clinical nurses, be dynamic, the student will be encouraged to be dynamic and innovative. If the system moves constantly, it is actually alive, like flowing water that can be life-giving. (Female, 2nd semester PhD student, 34 years old)*

#### Existing from self-made fence

From the participants’ point of view, a student was innovative if tried to break through barriers such as routines and mental and functional frameworks and sought new paths. The subcategories were escape from daily routine, intellectual and practical independence, and up-to-date nursing knowledge.

Regarding escape from daily routine, the participants emphasized issues such as acting out of routine, variety in the way of doing things, and trying new ways of learning, thinking, researching, and taking care of patients.

*If I want to define the concept of innovation in the field of nursing, it means moving in the opposite direction, the direction that everyone is going. In the major of nursing, all the last semester students start working or studying for a master’s degree, and this is the same for all the nursing students. Acting out of these routines means being innovative. (Male, 4th semester graduate student, 27-year-old)*

One of the participants in the field of intellectual and practical independence said:

*“The first characteristic that an innovative student must have is independence, the probability of experiencing innovation by a dependent person is one% or one in a thousand, but when he/she has independence, a task is left to him/her, he/she thinks that he/she will make ideas and start to be innovate.” (Male 5th semester undergraduate student, 22 years old)*.

The participants also emphasized the importance of updating nursing knowledge. In the regard one of the participants said:

*“Innovation means that a person has sufficient and up-to-date knowledge in relation to various clinical subjects, etc… This causes a spark in his/her mind that there is still something that is not working or has received less attention. As long as there is no up-to-date knowledge, there will be no individual innovation.” (Male, 5th semester graduate student, 25-year-old)*.

#### Structural and process change

From the participants’ point of view, transformation, a new change or one in the direction of improving a process or product, was one of the main components of innovation. Participants also considered adaptation to change as an innovation. Accordingly, the two sub-categories of change and transformation in the processes and system of nursing and individual and professional adaptation and coping with change formed this category. One of the participants regarding change in the nursing system and processes said:

*The first thing that comes to my mind from the word of innovation is that a person decides to make a change to improve a process or a product, … that no one else has been able to create before. (Female, 4th semester PhD student, 34 years old)*

Regarding adaptation and coping with a change, one of the participant said:

*“A part of innovation is how we can adapt to changes. For instance, due to COVID 19, quarantine deprives you of many things, but many people have many innovations during this quarantine period, so one dimension of innovation is how we actually adapt ourselves to a change.” (Female, 7nd semester PhD student, 37 years old)*.

### Professional inventiveness

Another important component in defining individual innovation in a nursing student was professional inventiveness which itself included two categories of intellectual inventions in nursing and practical inventions in nursing.

#### Intellectual inventions

>From the participants’ point of view, innovators are always looking for new approaches with new functions in their way of thinking. A participant said:

*Innovation itself means having a series of intellectual creativity to find unknown things, or to find something that has not been worked before, or things that have received less attention. Or we can say intellectual creations to solve a series of issues or a series of views and theories. (Male, 5th semester graduate student, 25-year-old)*

#### Practical inventions in nursing

One of the students spoke about practical inventions in nursing:

*There is another kind of innovation, which is to invent something. For example, to combine two devices, or to create something with a completely new function. I feel in our field doing things in a new way is a kind of innovation. (Male 5th semester undergraduate student, 22 years old)*

### Integration of innovation drivers

Participants emphasized that many factors could facilitate or inhibit innovation in nursing students. To bring up an innovative student, the drivers of innovation must be put together and aligned, and efforts must be made to reduce or eliminate barriers against innovation. Accordingly, 4 categories of mental, cognitive, and psychological capabilities, internal stimuli, external stimuli, and support networks formed this theme.

#### Mental, cognitive, and psychological capabilities

From the participants’ point of view, innovative nursing students had abilities and characteristics that differentiated them from others and made them an innovative person. These competencies fell into the ten subcategories of thinking, exploring and questioning, deepening insight, self-confidence, perseverance, risk-taking, problem-solving skills, clinical decision-making skills, inter-professional and teamwork skills, and leadership power. Regarding an innovative student’s capabilities, including thinking, perseverance, and risk-taking, one of the participants said:

*I think an innovative student is more thoughtful than others, more concerned than others … She/he thinks and is looking for a new way to solve problems, and even though she/he knows she/he may suffer from many damages, she/he steps in this direction. It is very valuable that with all this fear, threat, and ridicule, she/he continues her/his way… We can say that she/he has perseverance and takes risks. (Male, 4th semester graduate student, 27 years old)*

Another factor facilitating innovation, which was emphasized by many participants, was inter-professional and teamwork skills. As one said:

*“Another important issue about innovation is that innovation is a team and inter-professional work, that is, if a person innovates alone, he/she cannot carry out the work, he/she must be able to form a team of people with different expertise so that he/she can promote the innovation he/she has in his/her mind and make it a reality.” (Female, 4th semester PhD student, 34 years old)*.

#### Internal stimuli

In addition to the above-mentioned characteristics, the participants considered the internal stimuli as other factors affecting innovation, comprising of 4 subcategories of individual desire and interest, meeting individual needs, belief in the possibility of innovation in nursing, and having talent for innovation. One of the issues mentioned in the subcategory of personal desire and interest was the student’s interest in the field of nursing.

*I saw a lot of students that had no interest in nursing, so how can they be innovative? In my opinion, individual interest in nursing, which plays an important role in the care and patients’ recovery’, is very important in individual innovation. (Male, 5th semester PhD student, 32 years old)*

Regarding meeting individual needs, one of the participants said:

*Innovation can be used to meet the individual’s needs. For example, one type of innovation may be made in the way of studying, where everyone chooses a method according to their needs. My way of studying may be different from that of my friend because we have different needs. (Female, 6th semester undergraduate student, 20 years old)*

Considering belief in the possibility of innovation in nursing, one of the participants said:

*“Innovation in nursing can definitely happen, you definitely have to believe that it will happen, and this will cause an innovation that may not be notable, but at least that innovation can alleviate some problems or make some nursing issues easier.” (Male, 5th semester undergraduate student, 22 years old)*.

With respect to having talent for innovation, one of the participants said:

*“The talent for innovation is also important, in my opinion, every person can be creative and innovative according to their innovative talent and abilities as long as they want to be innovators.” (Female, 7nd semester PhD student, 37 years old)*.

#### External stimuli

In addition to individual characteristics and internal stimuli, external stimuli were also one of the important issues raised by the participants. External stimuli included 4 subcategories of motivation and motivators of ideation, meeting the needs of the profession and society, teachability of innovation, and creating an environment for discovery and creation. The motivation and motivators of ideation included the three infra-categories of motivation, effective and purposeful feedback, and respect for the student and his or her new proposals.

One of the master’s students described the effect of lack of motivation on individual innovation in nursing students as follows:

*“Unfortunately, many students do not have enough motivation for innovation in nursing, while they have very good thoughts and ideas, they work in various other fields, and many of them work in art fields. They can use these ideas and thoughts in different fields of nursing. Therefore, there must be motivation for a person to move towards innovation.” (Male, 5th semester graduate student, 25-year-old)*.

The last subcategory of external stimuli was mentioned by almost all participants. This subcategory included the infra-categories of self-fulfillment, identifying innovative students, purposeful interaction with innovators, cultivating and developing students’ reasoning and thinking power, active teaching and learning, employing qualified professors, and improving reforming the university environment, the family and social environment, the clinical environment, and ultimately the bureaucracies and policies in the field of nursing.

*A person who enters the university, the university environment and the people around her/him affect her/his innovation. Her/his friends and professors and even the community he/she has entered are very important, because they may make her/him thinking much deeper. So the academic and educational environment and the professors as a whole are very influential and should be in a way that contributes to the flourishing of innovation. (Male, 5th semester graduate student, 25 years old)*

#### Support networks

According to the participants’ view, the support received from nursing policy makers, family, community, university, and clinical settings was the other factor influencing nursing students’ innovation. This category consisted of the subcategories of supportive policies, university support, family and community support, quality and quantity of access to equipment and financial facilities, and quality of access to individuals and innovation guidance centers. The support of the university was emphasized by many participants. In this regards, one of them said:

*“For example, I had many ideas about nursing care apps, but no one supported me in the university. Such innovations must be supported so that a person can start. In the university, no one supported meand Everyone said that you should do it yourself, you should start by yourself.” (Female, 1th semester graduate student, 29-year-old)*.

Regarding the support of the managers, one of the participants said:

*No innovation will be formed in the student unless the nursing managers, whether in universities or hospitals, support creative and innovative students, and students who really have new and creative ideas. (Female, 2nd semester PhD student, 34 years old)*

## Discussion

This study explored the concept of individual innovation from the perspective of nursing students. From the participants’ point of view, personal and professional dynamism was one of the components of innovation. A review study also showed the positive effect of dynamism on innovative practices [33]. In addition, innovation can maintain the dynamism of nursing education [34]. In contrast to the present study, Neiboer et al., (2012) in the Netherlands showed an inverse relationship between environmental dynamics and innovative culture in long-term care centers. The reason for this inverse relationship could be changes in the Dutch health care system aiming to increase the quality of care but have reduced the culture of innovation in delivery of care services [35].

In addition to dynamics, exiting the self-made fence was another component of personal and professional dynamism. According to previous studies, nursing leaders can encourage the promotion of innovation by providing opportunities for nurses’ autonomy [36, 37]. In addition, provision of independence can affect nurses’ innovative behaviors [38]. Therefore, it seems that independence and overcoming the barriers could be other components of nursing student innovation.

Based on the findings of the present study, structural and process change was another component of personal and professional dynamism. According to previous studies, environmental dynamics is characterized by rapid changes in production, accelerating the innovation [35]Hurt (1977) considered the tendency to change as the most correct interpretation of innovation [29]. Innovation can involve fundamental but not necessarily revolutionary changes. Recently, acceptance of change, including new services, ideas, and new way of doing things are interpreted as innovation [39]. Therefore, in addition to the need for innovation to bring about change and transformation, innovation also makes a system or organization adaptable to changes [40]. In the present study, in addition to making change, the participants also considered adaptation to change as an innovation.

According to the results of the present study, one of the dimensions of innovation was inventiveness in nursing. According to Ackerman (2021), the core of innovation is the creation of new ideas or the use of existing ideas in new ways or in new situations. He also believes that nursing leaders need activities to create insights, culture, structure, and methods to apply new ideas in nursing practice [41]. According to the present study, innovation in nursing can include intellectual and practical inventions in nursing. Also, based on a concept analysis on innovative behavior in nursing context, idea generation and application are two of the attributes to innovative behavior in nursing [42]. Literature published from 1960 to 1980 also considered innovation inherently as a new idea emerged from some descriptions (intellectual invention). In addition, innovation can be defined as application of creative and new ideas and inventions (practical invention) [39]. Based on the findings of a previous study, an innovative person not only has the ability to create new ideas and intellectual invention, but also has the ability to turn ideas into new products or services and create practical invention [43]. Invention is the second state of knowledge that is something not previously demonstrated to be possible in practice. Key attributes of invention are feasibility and novelty. Innovation as the third state of knowledge means that the invention reaches the final stage, which can be a practical device, service or product offered in mass production, and actually it is the stage of implementation of an invention in society and market. The key attribute of innovation is utility, in addition to the novelty and feasibility of the invention [44]. Fagerberg defines invention as “the first occurrence of an idea for a new product or process, while innovation is the first attempt to carry it out into practice.” [45]. Based on the results of the present study, inventiveness in nursing can be the foundation of individual innovation in nursing students.

Based on the results of the present study, mental, cognitive, and psychological capabilities of the individuals, internal stimuli, external stimuli, and support networks were the four categories of integration of innovation drivers that, if aligned, could make innovation in nursing students possible.

From the participants’ point of view, mental, cognitive, and psychological capabilities, such as thinking, exploration and questioning, deepening insight, self-confidence, perseverance, risk-taking, problem-solving skills, clinical decision-making skills, inter-professional and teamwork skills, and leadership power, can be the characteristics of an innovative nursing student. Previous studies have also supported the relationship between personal characteristics and innovative outcomes and creativity. In the case of concepts close to innovation, such as creativity, the psychological, cognitive, and mood status of individuals has been a direct predictor of creative performance and modifier of the underlying factors affecting such performance [43]. Based on a concept analysis, individual characteristics is one of the antecedents to innovative behavior in nursing context [42]. Other studies on nurses or nursing students also indicate the relationship between characteristics, such as questioning, insight, self-confidence, leadership, perseverance, and risk-taking, and innovation [37, 39, 46, 47]. Thus, it seems that individual characteristics affect people’s innovation [43], and psychological empowerment of individuals can influence innovative behaviors [41]. Therefore, it can be necessary for those involved in nursing education to try to develop innovation-enhancing characteristics in students and design appropriate plans and interventions in this regard.

According to the results of the present study, internal and external stimuli were other factors affecting innovation. Similarly, Fisher et al., (2019) indicated that internal stimuli had a positive effect on innovation and external stimuli modulated the relationship between internal stimuli and innovation [48]. In Shahsavari Isfahani et al.’s study (2015), internal and external stimuli were also among the stimuli of creativity in clinical nurses and in line with the present study, it was concluded that interest in nursing was one of the most important internal stimuli [49]. According to a systematic review, internal stimuli, such as interest, had a greater impact on innovation than external stimuli [50]. In addition, it was found that interest increased satisfaction and the ability to innovate and perform a challenging task [49]. From the perspective of emotion theories, internal stimuli also increase psychological interaction and energy [51]. According to self-determination theoretical view, it is possible to encourage people to do challenging, complex, and unfamiliar tasks through reinforcing their interest and internal stimuli [52]. Therefore, to flourish the innovation of nursing students, nursing education systems, especially in Iran, should help nursing students identify their areas of interest and set precise criteria, such as interviews, to select more interested students in nursing [49]. Among the external stimuli, almost all participants placed great emphasis on the impact of the environment on the emergence of innovation, which included the educational, clinical, family, social, and work, and policy environments. The results of other studies also indicated the important role of work, organizational, clinical, and policy environments in involving people in innovative activities [38, 39, 43]. It seems innovative atmosphere encourages people to enter innovative activities, facilitates the development of effective functional approaches, and enhances quality of care [46]. Nursing managers and leaders must provide the appropriate environment to promote innovation by providing opportunities and implementing suitable educational activities and legislation [36, 38]. In addition, innovation must be an integral part of all strategies and policies [39].

From the perspective of the participants in the present study, support networks were another driver of innovation. Zappala (2021) also showed the positive effect of supporting innovation on the use of ideas [37]. However, a study consistent with the present study yielded that nurses felt lack of support to accept the risk of innovation and that leaders could not provide their full support in this regard [47]. According to a qualitative study, organization’s support of innovators is crucial [19], and nursing leaders and managers as well as educational and clinical organizations should provide sufficient financial, supervisory and colleague’s support to encourage individuals to participate in innovative activities [39, 43], so that they feel confident that their creative and innovative approaches are valuable for problem solving [40]. On the other hand, designing interventions to support innovation can lead to growth and self-actualization of individuals [46].

Hence, it is necessary to pay attention to the concept of innovation in nursing students who will be at the forefront of treatment as nurses in the future and can provide innovative solutions to current and future problems of the health care system [39]. One of the problems in Iran is that innovation is not considered a part of formal education and professional socialization. Innovation, on the other hand, is an evolving, systematic, and multi-factor process that is influenced by individual, group, and organizational factors, and it is unlikely that fate, divine intervention, or chance leads to innovation [19, 37, 39]. It is necessary to pay attention to innovation and its management in education and clinical settings [19].

The small number of studies investigating the individual innovation among nursing students was one of the limitations of the present study. Another limitation of the present study was conducting some interviews via WhatsApp video call or telephone due to the conditions caused by the COVID-19 pandemic and lack of access to some students. However, we tried to overcome this limitation through conducting additional interviews with participants. Data collection from only one nursing school in southern Iran was another limitation of the present study. Therefore, more extensive studies are recommended considering these limitations by resorting to other research approaches such as combined quantitative and qualitative research to increase knowledge about the concept of innovation in nursing students.

## Conclusion

From the participants’ point of view, the concept of individual innovation in a nursing student included three main themes of personal and professional dynamism, professional inventiveness, and the integration of innovation drivers. This study provided a comprehensive understanding of the concept of individual innovation in nursing students and a clearer definition for this concept. Managers and policy makers of nursing education can use the results of this study to get acquainted with the concept of individual innovation in nursing students and the factors affecting it and accordingly develop policies and guidelines for the development of individual innovation in nursing students and take steps to promote student’s innovation using appropriate interventions. Managers can also take positive steps to pave the way for innovation in nursing students by considering the internal and external drivers of innovation. Nursing students can also try to flourish their innovation by becoming familiar with the definition of individual innovation and its components. In the future, as innovative nurses, they will enter nursing practice, management, education, and research and lay the groundwork for tremendous changes in health care and nursing.

## Electronic supplementary material

Below is the link to the electronic supplementary material.

Supplementary Material 1

## Data Availability

The datasets analyzed during the current study are available from the corresponding author on reasonable request.
